# Does Intention Strength Moderate the Intention–Health Behavior Relationship for Covid-19 Protection Behaviors?

**DOI:** 10.1093/abm/kaad062

**Published:** 2023-10-24

**Authors:** Mark Conner, Sarah Wilding, Paul Norman

**Affiliations:** School of Psychology, University of Leeds, Leeds, UK; School of Psychology, University of Leeds, Leeds, UK; Department of Psychology, University of Sheffield, Sheffield, UK

**Keywords:** Behavioral intention, Intention strength, Temporal stability of intention, Intention–behavior gap, Goal priority, Goal conflict

## Abstract

**Background and Purpose:**

The present research tests whether intention strength moderates intention–health behavior relations and the extent to which this is accounted for by the moderating effects of intention stability, goal priority, and goal conflict.

**Methods:**

In a prospective multi-behavior study, a representative sample of UK adults (*N* = 503) completed measures of past behavior, intention, intention strength, goal priority, and goal conflict in relation to eight Covid-19 protection behaviors at time 1. Intention and self-reported behavior were assessed at time 2 (2 months later). Intention stability was assessed over 2 months.

**Results:**

Intention strength was a significant moderator of the intention–behavior relationship (controlling for past behavior). Controlling for the moderating effects of intention stability attenuated the moderating effect of intention strength, while also controlling for the moderating effects of goal priority and goal conflict reduced the moderating effects of intention strength to nonsignificance.

**Conclusions:**

The present findings indicate that intention strength is a significant moderator of the intention–health behavior relationship. They also suggest that the moderating effect of intention strength is explained by effects on intention stability, goal priority, and goal conflict. Tests of interventions to manipulate intention strength as a means to strengthen intention stability and intention–behavior relations are warranted.

## Introduction

Behavioral intention (e.g., I intend to eat at least 5 portions of fruit and vegetables each day) is a key proximal determinant of engaging in behavior that is included in a range of theories used to predict health behaviors (e.g., Theory of Planned Behavior [1], Protection Motivation Theory [2], and Social Cognitive Theory [3]). Various meta-analyses indicate behavioral intention to be one of the strongest predictors of a range of health behaviors (e.g., [4]). Nevertheless, such reviews also indicate that intention only explains a minority of the variance in health behaviors (18% in [4]). This disjunction between intention and behavior has been termed the intention–behavior gap ([5]; see also [6, 7]). Although a range of methodological factors are associated with either a narrowing or widening of the intention–health behavior gap (see [8, 9] for reviews), it is theory-based moderators of this relationship that have the greatest potential to improve understanding. The current paper focuses on the concept of intention strength in furthering our understanding of the intention–health behavior gap.

Variables associated with changes in the magnitude of the intention–health behavior relationship (i.e., moderators) have been a focus of work on the intention–behavior gap for a number of years (e.g., [5, 9, 10]). Moderators help identify the limits of the relationship between intention and behavior and also the operating conditions [11] under which strong versus weak relationships might be expected (i.e., when the intention–behavior gap may be smaller or larger). For example, a recent review by Rhodes et al. [10] on moderators of the intention–physical activity relationship identified 129 studies with 138 independent samples. Moderators tested included 19 different sociodemographic and/or medical variables, 7 personality variables, 5 physical capability variables, 10 psychological capability variables, 5 social opportunity variables, 9 environmental opportunity variables, 6 automatic motivation variables, and 17 reflective motivation variables. Temporal stability of intentions was the most consistent moderator identified in this review (significant in 9/12 [75%] tests).

The current research extends this work by drawing on the theoretical concept of *intention strength* as an additional moderator of the intention–behavior relationship that has been little tested in the health domain. Intention strength [8] parallels the attitude strength [12] concept. Conner and Norman [8] argue that intention strength can be defined in relation to impactfulness, that is, strong intentions should be predictive of behavior and guide information decision making processes, and durability, that is, strong intentions should be stable over time and resistant to change. In the present context, it is suggested that strong intentions should be more predictive of behavior, in part, because they exhibit temporal stability.

Conner and Norman [8] drew parallels with the attitude strength literature to argue that there might be a number of underlying subcomponents to, or predictors of, intention strength. For example, recent reviews of attitude strength [13, 14] identified attitude certainty, importance, moralization, elaboration, and knowledge (in addition to extremity), as key subcomponents of attitude strength. Research has shown each of these subcomponents to moderate the attitude–behavior relationship [15]. However, analyses support the idea that they are typically strongly intercorrelated and often form a single latent variable [16, 17].

Conner and Norman [8] argued that intention strength might also consist of the same five, distinguishable subcomponents. Intention *certainty* refers to the degree of confidence an individual has that his or her intention is correct/clear to him or her. A limited number of studies have shown intention certainty to be a significant moderator of the intention–behavior relationship [5, 18–22]. *Moralization* or moral conviction is the degree to which the intention reflects a strong and absolute belief that something is right versus wrong, moral versus immoral, or that it reflects core moral values and convictions [15, 23, 24]. In relation to intentions, Godin et al. [25] showed that intentions more closely aligned with moral norms (compared with attitudes) were more predictive of subsequent behavior. Other subcomponents of intention strength have not been tested as intention–behavior moderators. Intention *importance* refers to the degree to which an individual attaches significance to the intention or behavior. Although this has been shown to be a key component of attitude strength [13] and shown to moderate the attitude–behavior relationship (e.g., [15]), it has not previously been tested in relation to the intention–behavior relationship. Intention *knowledge* refers to the amount of information the individual has about the behavior (i.e., knowledge volume). Greater attitude knowledge has been found to be associated with stronger attitude–behavior relationships [15, 26]. Relatedly, *elaboration* is the degree of thought or careful consideration one has given to the merits and shortcomings of a behavior [27]. Studies have shown more elaborated attitudes better predict behavior [28]. To date there have been no published tests of intention knowledge or intention elaboration as moderators of the intention–behavior relationship.

Although the above five subcomponents of intention strength may be theoretically distinguishable, in practice they are likely to be highly intercorrelated, forming a single latent variable [8]. In the current research, the focus was on the overall construct of intention strength (tapped by these five subcomponents) as a moderator of the intention–behavior relationship. The degree of intercorrelation of the subcomponents and the extent to which in measurement terms they formed a single latent variable tapping overall intention strength was also examined.

### Explanatory Mechanisms

In addition to examining intention strength as a moderator of intention–behavior relations, the current research further focused on examining the mechanisms through which such an effect might occur. In particular, the focus was on intention stability and goal properties (i.e., goal priority and goal conflict) that might account for any moderating effects of intention strength.

Conner and Norman [8] in their discussion of intention strength noted that as well as being more predictive of behavior, strong intentions are also likely to have greater temporal stability. In relation to the current research, the temporal stability of intention might help to account for any moderating effects of intention strength on the intention–behavior relationship, that is, strong intentions are more predictive of behavior because they are more stable. The temporal stability of intention has long been suggested as an important mechanism through which intention better predicts behavior [29] with consistent empirical support (see, e.g., [10] for a review). Thus, the temporal stability of intentions may be a key mechanism through which other moderators of the intention–health behavior relationship may have their effects. Sheeran and Abraham [22] is one of the few studies to show intention stability fully explained the moderating effects of several intention–behavior moderators (i.e., certainty, past behavior, self-schema, anticipated regret, attitudinal control) for the intention–physical activity relationship. To date, there have been no studies testing whether intention stability explains the moderating effect of intention strength on the intention–behavior relationship. This was a focus here.

Goal constructs represent a second potential mechanism to account for intention strength as a moderator of the intention–behavior relationship. That is, stronger intentions may lead to changes in goal properties (e.g., the extent to which they are prioritized and are perceived not to be in conflict with other goals) that themselves moderate the intention–behavior relationship. Thus, goal priority and goal conflict are the two goal constructs that have been most examined as moderators of the intention–behavior relationship [8]. *Goal priority* refers to the temporary increase in the importance attached to, and resources directed toward, one or more goals compared with other goals—that serve to benefit the performance of the prioritized behavior [30]. Goal priority has received attention as an intention–health behavior moderator [31, 32]. For example, Conner et al. [31] showed that goal priority moderated the intention–behavior relationship for physical activity (Study 1) and a range of health behaviors (Study 4). *Goal conflict* taps the degree to which a focal goal conflicts with other goals. Less goal conflict might be expected to be associated with greater effort to achieve the focal goal and so stronger intention–behavior relationships. Rhodes et al. [10] reported that goal conflict significantly moderated the intention–physical activity relationship in approximately 70% (6/9 tests) of studies reviewed. Although goal priority and conflict could form a general goal construct, most research indicates only modest correlation between the two [31]. To date, there have been no studies testing whether goal priority and conflict account for the moderating effect of intention strength on the intention–behavior relationship. This was also a focus here.

In summary, the current research tests a general measure of intention strength (tapped by five subcomponents) as a moderator of the intention–health behavior relationship in a study assessing multiple Covid-19 protection behaviors. The effects of past behavior were controlled for as it is one of the strongest predictors of future behavior and also provides an indication of the sufficiency of a model. In addition, we assessed the power of intention stability to explain the moderating effects of intention strength on the intention–behavior relationship [8, 22]. Finally, we assessed whether two key goal properties (goal priority and goal conflict) previously considered as moderators of the intention–behavior relationship [8, 10] represented additional mechanisms to explain any moderating effect of intention strength on the intention–behavior relationship.

The above predictions were tested in relation to eight Covid-19 protection behaviors that have been recommended by the World Health Organization [33]. These behaviors were selected to test our predictions for two reasons. First, identifying the key predictors of Covid-19 protection behaviors represents an important and topical issue. Moreover, the rapidly changing nature of the Covid-19 pandemic (e.g., changes in guidelines, vaccination rates, etc.) provides an ideal context in which to test effects of intention strength and intention stability compared with many other health behaviors that are conducted in relatively stable contexts. Second, intention has been found to be a key predictor in many studies examining Covid-19 protection behaviors, including physical distancing [34–36] and hand washing [36], as well as multiple Covid-19 protection behaviors [37–39] such as are examined here. Across these studies intention has been found to explain between 11% and 37% of the variance in Covid-19 protection behaviors, figures comparable to the 18% reported in a meta-analysis of the theory of planned behavior applied to health behaviors more generally [4]. Although significant, such values indicate a considerable portion of the variance in behavior remains unexplained by intentions. In addition, several studies [37, 40] have shown intention stability to moderate the intention–behavior relationship for Covid-19 protection behaviors, although none to date have focused on the moderating role of intention strength.

## Method

### Participants and Procedure

Prolific (www.prolific.co) was used to recruit a representative sample of UK adults (in relation to age, sex, and ethnicity) via stratified sampling. Eligible individuals from the Prolific participant pool were invited to take part in a study on Covid-19 protection behaviors. After reading an information sheet, individuals clicked on a number of statements to provide informed consent to participate. Participation comprised of completing two online surveys hosted on Qualtrics 2 months apart (time 1, November 30, 2021; time 2, January 31, 2022). This was also a period when UK cases rates and deaths from Covid-19 were still high (67.5k cases per day; 0.2k deaths per day) and some restrictions were in place linked to the use of face coverings. Ethical approval for the study was granted by University of Sheffield, UK Research Ethics Committee (ref. 044118).

A total of 503 and 445 participants completed the time 1 and 2 surveys, respectively. Of the baseline sample, 85.1% reported that they had been diagnosed with or had received a positive Covid-19 test and 66.8% reported that they had self-isolated. In addition, 87.5% reported that they had been vaccinated at least once. The baseline sample was broadly representative of the UK adult population in relation to sex (females: 50.6% vs. 51.1%) and ethnicity (ethnic minorities: 15.0% vs. 19.5%) but slightly biased toward younger participants (18–24: 12.0% vs. 14.6%, 25–34: 17.0% vs. 27.2%, 35–44: 17.7% vs. 22.0%, 45–54: 17.6% vs. 18.0%, 55+: 35.7% vs. 17.8%) (UK vs. study sample [41]). Those who were lost to follow-up were younger (*M* = 34.64) than those who completed both surveys (*M* = 40.75), *t*(497) = 3.10, *p* = .002. However, they did not differ in terms of sex, χ^2^(1, *N* = 503) = 0.03, *p* = .859, or ethnicity, χ^2^(1, *N* = 503) = 0.36, *p* = .549.

### Measures

The measures included items assessed in relation to performing each of eight Covid-19 protection behaviors recommended by the World Health Organization [33] over the next 2 months: Wearing a face covering in public places; Maintaining social distancing of at least 1 meter; Hand sanitizing regularly; Avoiding the three “Cs” (Closed spaces, Crowded places, and Close contact); Cleaning surfaces regularly; Covering your mouth/nose when coughing/sneezing; Meeting people outside rather than indoors; Opening a window to increase natural ventilation.

*Intention* was assessed at both time 1 and 2 by three items for each behavior (e.g., “Do you intend to wear a face covering in public places in the next two months? Definitely don’t–Definitely do”; “I will try to wear a face covering in public places in the next two months, Definitely won’t–Definitely will”; “I expect to wear a face covering in public places in the next two months, Definitely no–Definitely yes”; all scored 1–7; *a*’s = 0.93–0.97; items averaged for each behavior). *Intention stability* between time 1 and 2 was assessed as 6 minus the absolute difference between time 1 and 2 intention measures for each behavior, with high scores indicating greater temporal stability [37, 42].

Five *intention strength* measures were assessed at time 1. *Certainty* was assessed as a single-item focusing on the intention (e.g., “How certain are you of your intention to wear a face covering in public places?, Not at all certain–Extremely certain”; scored 1–7) for each behavior. Single-item measures were used to tap *importance* (e.g., “How important is wearing a face covering in public places to you? Not at all important–Extremely important”; scored 1–7), *moralization* (e.g., “Morally, wearing a face covering in public places is the right thing to do? Strongly disagree–Strongly agree”; scored 1–7), *knowledge* (e.g., “How much do you know about the reasons/evidence for why you should wear a face covering in public places? Not much–A lot”; scored 1–7), and *elaboration* (e.g., “How much thought have you given to whether or not to wear a face covering in public places? No thought–A lot of thought”; scored 1–7) for each behavior. Factor analysis indicated a single factor explaining 60.6% of the variance. These items were therefore averaged (equal weighting) to form a single construct of intention strength (α = 0.82).

Two goal properties were also assessed at time 1. Single-item measures for each behavior were used to tap *goal priority* (“I will prioritize wearing a face covering in public places in the next two months over other goals important to me, Strongly disagree–Strongly agree”; scored 1–7); *goal conflict* (e.g., “Wearing a face covering in public places in the next two months would conflict with other goals important to me, Strongly disagree–Strongly agree”; scored 1–7). Goal priority and goal conflict were unrelated (*r* = −.03).

*Behavior* of the each of the eight Covid-19 protection behaviors was assessed at time 1 and 2 with two questions: engagement with the behavior (e.g., “To what extent have you done each of the behaviors listed below over the past month? Not at all–All the time”; scored 1–7); engagement with the non-protection behavior over the past month (e.g., “Not worn a face covering in public places? Not at all–All the time”; scored 1–7. Based on the skewed responses, the two items were combined to produce a dichotomous measure for each behavior: scores of 7 for engagement with protection behavior *and* 1 for engagement with non-protection behavior were coded fully compliant (scored 1); all other patterns of responses were coded non-fully compliant (scored 0) [38]. The time 1 measure was used to tap past behavior.

Only items relevant to the current research are reported here (the full questionnaire can be obtained from the first author).

### Analyses

Analyses were conducted in SPSS (version 24, SPSS Inc.) and HLM (version 7, SSI). Participants with missing data for at least one variable for each behavior were excluded from the main analyses (i.e., listwise deletion). Correlations and means and standard deviations across behaviors were assessed first. The main analyses used multilevel analysis to take account of eight behaviors being measured within each participant [15, 38, 39]. Multilevel regression analyses were used to assess intention strength, intention stability, goal priority, and goal conflict as moderators of the intention–behavior relationship, that is, to test the significance of the interactions between intentions and each moderator.

In Model 1, past behavior, intention, intention strength, and the intention × intention strength interaction were entered as predictors of behavior at time 2. In Model 2, intention stability and the intention × intention stability interaction were added. In Model 3, goal priority and the intention × goal priority interaction plus goal conflict and the intention × goal conflict interaction were added. Where interactions were significant (*p* < .05), indicating a moderation effect, the direction of effect was established with simple slopes using the Preacher free software (level 1 interactions: http://www.quantpsy.org/interact/hlm2.htm). For all the regression analyses, model fit (−2 log likelihood for the Bernoulli regressions predicting behavior) is reported along with unstandardized coefficients, standard errors, odds ratios, and significance (based on the population-average model with robust standard errors) for each predictor.

## Results

Table 1 reports mean and standard deviation plus intercorrelations among the key variables. Intention, intention strength, intention stability, past behavior, and goal priority were each significantly positively correlated with behavior at time 2, while goal conflict had a significant negative correlation. Intentions were also significantly positively correlated with intention strength, intention stability, past behavior, and goal priority, but negatively correlated with goal conflict. Intention strength had significant positive correlations with intention stability, goal priority, and past behavior, and a significant negative correlation with goal conflict. Intention stability had significant positive correlations with goal priority and past behavior and a significant negative correlation with goal conflict.

**Table 1:** Means, Standard Deviations, and Intercorrelation of Measures (*N* = 3,559)

	B_T2_	BI	BISTB	BISTR	GP	GCON	PB
Behavior (B_T2_)	1.000						
Behavioral intention (BI)	0.445^***^	1.000					
Intention stability (BISTAB)	0.329^***^	0.171^***^	1.000				
Intention strength (BISTR)	0.424^***^	0.828^***^	0.226^***^	1.000			
Goal priority (GP)	0.370^***^	0.728^***^	0.182^***^	0.726^***^	1.000		
Goal conflict (GCON)	−0.148^***^	−0.202^***^	−0.071^***^	−0.092^***^	−0.029	1.000	
Past behavior (PB)	0.600^***^	0.446^***^	0.310^***^	0.439^***^	0.379^***^	−0.161^***^	1.000
*M*	0.281	5.471	5.054	5.192	4.631	3.021	0.247
*SD*	0.450	1.776	1.118	1.342	2.031	2.105	0.431

^***^ *p* < .001.

The regressions reported in Table 2 showed that past behavior, intention, intention strength, and the intention × intention strength interaction were each significant predictors of behavior (Table 2, Model 1). Simple slopes analyses indicated that although the relationship between time 1 intention and time 2 behavior was significant at all levels of intention strength, the size of the relationship increased as levels of intention strength increased from low (*M* − 1 *SD*: *B* = 0.227, *SE* = 0.028, *p* < .001) to moderate (*M*: *B* = 0.369, *SE* = 0.029, *p* < .001) to high (*M* + 1 *SD*: *B* = 0.511, *SE* = 0.038, *p* < .001).

**Table 2:** Moderated Hierarchical Regression of Behavior Onto Intention, Moderators, Interactions Plus Past Behavior (*N* = 3,559; 445 Participants)

Predictors	Model 1			Model 2			Model 3		
	*B*	*SE*	OR	*B*	*SE*	OR	*B*	*SE*	OR
Past behavior	1.661^***^	0.091	5.266	1.703^***^	0.102	5.492	1.597^***^	0.096	4.937
Intention	0.459^***^	0.033	1.583	0.353^***^	0.032	1.423	0.326^***^	0.032	1.385
Intention strength	0.135^**^	0.044	1.144	0.146^***^	0.043	1.157	0.102^*^	0.040	1.107
Intention × intention strength	0.126^***^	0.014	1.135	0.082^***^	0.016	1.086	0.021	0.018	1.022
Intention stability				0.286^***^	0.030	1.331	0.235^***^	0.026	1.265
Intention × intention stability				0.150^***^	0.017	1.161	0.162^***^	0.015	1.176
Goal priority							−0.016	0.021	0.984
Intention × goal priority							0.031^**^	0.011	1.031
Goal conflict							−0.036^**^	0.013	0.965
Intention × goal conflict							−0.021^***^	0.005	0.979

Note. For predictions of behavior, Model 1: −2 log-likelihood = 4,945.8; Model 2: −2 log-likelihood = 4,486.3; Model 3: −2 log-likelihood = −4,472.4.

^*^ *p* < .05.

^**^ *p* < .01.

^***^ *p* < .001.

Table 2, Model 2 showed that when added to the model, intention stability, and the intention × intention stability interaction were also significant. Past behavior, intention, intention strength, and the intention × intention strength remained significant in this model, although the size of the effect for this interaction was attenuated. Simple slopes analyses confirmed that although the relationship between time 1 intention and behavior was significant at all levels of intention stability, the size of the relationship increased as levels of temporal stability increased from low (*M* − 1 *SD*: *B* = 0.315, *SE* = 0.024, *p* < .001) to moderate (*M*: *B* = 0.530, *SE* = 0.020, *p* < .001) to high (*M* + 1 *SD*: *B* = 0.745, *SE* = 0.030, *p* < .001).

Table 2, Model 3 showed that when added to the model, goal priority and goal conflict were significant moderators of the intention–behavior relationship. Past behavior, intention, intention strength, intention stability, and the intention × intention stability interaction remained significant in this model. However, the intention × intention strength interaction became nonsignificant. Simple slopes analyses indicated that although the relationship between intention and behavior was significant at all levels of goal priority, the size of the relationship increased as levels of goal priority increased from low (*M* − 1 *SD*: *B* = 0.420, *SE* = 0.024, *p* < .001) to moderate (*M*: *B* = 0.483, *SE* = 0.029, *p* < .001) to high (*M* + 1 *SD*: *B* = 0.547, *SE* = 0.042, *p* < .001). Simple slopes analyses also indicated that although the relationship between time 1 intention and behavior was significant at all levels of goal conflict, the size of the relationship decreased as levels of goal conflict increased from low (*M* − 1 *SD*: *B* = 0.455, *SE* = 0.023, *p* < .001) to moderate (*M*: *B* = 0.406, *SE* = 0.018, *p* < .001) to high (*M* + 1 *SD*: *B* = 0.358, *SE* = 0.021, *p* < .001).

## Discussion

The current paper adopted an intention strength perspective to further our understanding of the intention–health behavior gap. This perspective views strong intentions as being durable and impactful, that is, strong intentions are stable over time and predictive of behavior [8]. The durability of strong intentions is viewed as one key mechanism behind the impact of strong intentions on behavior. The data reported here showed that our five measures of intention strength formed a single factor representing intention strength as a single overall latent variable. They also showed that intention strength significantly moderated the intention–behavior relationship, that is, when intentions were stronger they were better predictors of behavior. Supporting previous work (e.g., [10, 22]), intention stability was also shown to moderate the intention–behavior relationship, that is, more stable intentions were stronger predictors of behavior. More importantly the data indicated that part of the moderating effect of intention strength was attributable to intention stability (i.e., the moderating effect of intention strength is attenuated when controlling for the moderating effects of intention stability). In addition, the findings showed that the moderating effect of intention strength on the intention–behavior relationship was attenuated to nonsignificance when also controlling for the moderating effects of intention stability plus goal priority and goal conflict. Notably each of these three moderators of the intention–behavior relationship were significant when considered simultaneously with intention strength (Table 2, Model 3).

The current findings have both theoretical and practical implications. At the theoretical level, the focus on intention strength brings fresh insights to understanding the intention–health behavior gap by suggesting a new and under-researched moderator (i.e., intention strength) [8]. The current findings point to the value of focusing on intention strength as determinant of the intention–health behavior gap. In addition, targeting intention strength (see below) may be one useful way to change intention stability that has been identified as one of the most consistent moderators of the intention–health behavior relationship [10, 43]. This is important because there are few if any studies that have identified effective means to change intention stability. The findings also support other research showing that intention stability is an important mechanism explaining the effects of other intention–behavior moderators [22], including intention strength. Although one focus here was on intention stability, an intention strength perspective also highlights intention pliability and impacts on processing of intention-relevant information as other potential mechanisms to explain the moderating effect of intention strength on intention–behavior relations. Future research could usefully explore simultaneous effects of intention stability, pliability, and biases in information processing in this regard.

At the practical level, the present study suggests that targeting intention strength may represent an additional strategy in behavioral medicine’s toolbox for health behavior change. Interventions producing medium- to large-sized changes in intentions are associated with only small- to medium-sized changes in behavior [44]. Increasing the stability of intentions may increase the effectiveness of such interventions, given that intention stability is one of the most consistent moderators of the intention–health behavior relationship (see [10, 43] for reviews). However, there are few if any studies that have identified effective means to change intention stability. Targeting intention strength may therefore provide a novel route through which to increase intention stability. The present research does not identify which components of intention strength to target in an intervention, although intention certainty and importance might be useful targets. In relation to promoting Covid-19 protection behaviors in particular, the current research might suggest the value of targeting both intentions to engage in these behaviors, for example by targeting underlying behavioral, normative and control beliefs, plus intention strength, for example by increasing knowledge, or emphasizing the importance, of Covid-19 protection behaviors as means to increase intention stability.

There is a general lack of work on how best to change intention strength, although work on attitude strength and attitude certainty and importance in particular may provide useful clues. Tormala and Rucker [45] suggest that metacognitive appraisals drive people’s general feelings of uncertainty and, in particular, uncertainty about their attitudes. In particular, they highlighted the importance of appraisals about the accuracy, completeness, relevance, legitimacy, importance, and experienced ease of retrieval or use of attitudes. Tormala and Rucker [45] further suggest that manipulations focusing on consensus (i.e., others expressing similar views), repetition (i.e., repeating positive messages), ease of accessibility (i.e., repeated expression of the attitude), and defense (i.e., resisting a challenge to one’s attitude) may be useful ways to change these appraisals and so reduce attitude uncertainty. Similar manipulations may also be useful in relation to increasing intention certainty. For example, defending one’s plans to engage in a health behavior may increase the certainty with which intentions toward this behavior are held. In addition, Howe and Krosnick [13] suggest attitude importance is driven by self-interest, social identification, and values. In relation to importance of the behavior, this might suggest the value of messages targeting self-interest served by the health behavior (e.g., protecting your own health for the benefit of yourself and your loved ones), identifying with others who perform the health behavior, or linking the health behavior to one’s values as potential manipulations. Howe and Krosnick [13] also noted that public commitment to the attitude/behavior also increased importance.

The current research also showed that goal priority and goal conflict moderate the intention–behavior relationship. This supports a number of previous correlational (e.g., [31]) and intervention (e.g., [46]) studies. For example, Conner et al. [46] showed that writing down how to prioritize one or even two health behavior goals led to greater achievement of these goals without negatively affecting other health behavior goals that may have conflicted with the prioritized goals. The current findings also showed goal priority and goal conflict to help account for the moderating effect of intention strength on the intention–behavior relationship. This would suggest that strong intentions not only are more stable over time but also result in higher goal priority and less experience of goal conflict. However, it would be important for experimental studies to confirm whether the correlational relationships observed here translate into causal relationships. Experimental studies that independently manipulate each of our moderators (intentions strength, intention stability, goal priority, and goal conflict) would provide the strongest support for their independence. In addition, measurement studies that use confirmatory factor analysis to further establish the overlap of multi-item measures of each of these moderators would also be of value.

The current research has a number of strengths including the use of a large, nationally representative sample, examining effects across multiple moderators, controlling for past behavior, examining effects across multiple behaviors. It is also the first test of a general measure of intention strength as a moderator of intention–behavior relations. Nevertheless, there are also a number of weaknesses that should be acknowledged. First, the current findings need to be replicated using an objective measure of behavior. This is especially important given that meta-analyses indicate that intention explains more of the variance in self-report versus objective measures of behavior, although it is a significant predictor of both [4]. A second weakness was the reliance on single-item measures for some constructs (e.g., goal properties) that did not permit assessment of internal reliability, although it is worth noting that single-item scales have shown good predictive validity for assessing complex constructs such as self-esteem [47]. Moreover, we are not aware of specific evidence related to the current constructs to suggest that the use of single-item measures leads to systematic over- or underestimation of effect sizes. In addition, the current research employed meta-judgmental measures of intention strength. Operative measures of intention strength that rely less on introspection (e.g., knowledge quizzes to tap knowledge) may indicate more consistent effects [8]. Relatedly, although the current research assessed five subcomponents of intention strength, Conner and Norman [8] also identify accessibility as an additional intention strength measure, although the need to record speed of response might limit its applicability in survey studies. A third weakness is that our measure of intention stability used a measure of intention taken contemporaneously with our measure of behavior. This may have lead to an overestimation of the extent to which intention stability accounted for the moderating effect of intention strength. Previous research [37] has shown that intention stability significantly moderated the intention–behavior relationship whether or not intention stability was measured contemporaneously with behavior or whether behavior was measured at a later time point. A fourth weakness is the focus on just one set of behaviors (i.e., Covid-19 protection behaviors). Further research could usefully confirm whether the effects extend to other protection (e.g., behaviors in response to other infectious diseases and health threats) and risk (e.g., smoking, drinking alcohol) health behaviors. A final weakness was the reliance on correlational relationships. Future research that attempts to manipulate intention strength and then observe the effects on the intention–behavior relationship is needed to confirm that the correlational relationships observed here reflect causal processes.

## Conclusions and Future Directions

In conclusion, the current research shows the value of taking an intention strength perspective in helping to understand the intention–health behavior gap. Intention strength (as assessed by intention certainly, importance, moralization, knowledge, and elaboration) was a consistent intention–behavior moderator, with intention stability, goal priority, and goal conflict identified as potential mechanisms that explain its effects. Future research could usefully examine the impacts of manipulating intention strength on reducing the intention–behavior gap and testing the extent to which intention stability, goal priority, goal conflict fully mediates any effects.
